# Supplementary material for: Weiss or Wit: Chemical Profiling of Wheat Beers via NMR-Based Metabolomics
Source: Foods. 2025 May 3;14(9):1621. doi: 10.3390/foods14091621 (PMC12072173; doi:10.3390/foods14091621)
Supplement: Supplementary file 1 [file foods-14-01621-s001.zip › foods-3583784-supplementary.pdf]

Weiss or Wit: Chemical profiling of wheat beers via NMR–based metabolomics

Plamen Chorbadzhiev [1,2], Dessislava Gerginova [1], Svetlana Simova [1,*]

[1] Bulgarian NMR Centre, Institute of Organic Chemistry with Centre of Phytochemistry, Bulgarian Academy of Sciences, Acad. G. Bonchev str. Bl. 9, 1113 Sofia, Bulgaria; plamen.chorbadzhiev@orgchm.bas.bg (P.C.), dessislava.gerginova@orgchm.bas.bg (D.G.)

[2] Faculty of Chemical and Systems Engineering, University of Chemical Technology and Metallurgy, 8 St Kliment Okhridski blvd, 1756 Sofia, Bulgaria

* Correspondence: Svetlana.simova@orgchm.bas.bg (S.S)

SUPPORTING INFORMATION

LIST OF TABLES:

Table S1. List of the samples used - abbreviations, beer style, brewery, beer, alcohol by volume (ABV) and country of origin.

Table S2. Misclassification table of the OPLS–DA model distinguishing Hefeweizen, Weizenbock and Witbier.

Table S3. Misclassification table of the OPLS–DA model distinguishing Dunkelweizen, Kristallweizen and Hefeweizen.

LIST OF FIGURES:

Figure S1. Experimental (dark red) and deconvoluted (teal) ^{1}H NMR spectra of sample **wt10** shown across selected spectral regions: (a) 1.37–2.73 ppm, (b) 3.17–3.68 ppm, 4.44–5.20 ppm, and (c) 6.32–7.21 ppm, 7.62–9.70 ppm. Only signals used for quantitative NMR analysis were subjected to deconvolution.

Figure S2. ROC curve illustrating the OPLS-DA model's classification accuracy in distinguishing Hefeweizen, Weizenbock and Witbier.

Figure S3. ROC curve illustrating the OPLS-DA model's classification accuracy in distinguishing Dunkelweizen, Kristallweizen and Hefeweizen.

Figure S4. Permutation tests (25 iterations) assessing the statistical significance and robustness of two OPLS-DA models: (a) discrimination of three wheat beer styles – Hefeweizen, Weizenbock, Witbier and (b) differentiation of three wheat beer substyles – Dunkelweizen, Kristallweizen and Hefeweizen. The results confirm valid and non-random class separation in both models.

Figure S5. OPLS-DA contribution plots demonstrating the differentiation of significant compounds among Hefeweizen (blue), Weizenbock (red) and Witbier (yellow).

Figure S6. Nightingale diagrams illustrating the levels of metabolites in commercial (light blue) and craft (blue) Hefeweizen samples, categorized by compound class (alcohols, saccharides, organic acids, amino acids, nucleosides, and others).

Figure S7. Nightingale diagrams illustrating the levels of metabolites in commercial (yellow) and craft (brown) Witbier samples, categorized by compound class (alcohols, saccharides, organic acids, amino acids, nucleosides, and others).

Figure S8. OPLS-DA contribution plots demonstrating the differentiation of significant compounds among Dunkelweizen (black), Kristallweizen (purple) and Hefeweizen (blue).

Table S1. List of the samples used - abbreviations, beer style, brewery, beer, alcohol by volume (ABV) and country of origin.

Code	Style	Brewery and beer	Alcohol by volume, %	Country of origin
**dw1***	Dunkelweizen	Privatbrauerei Eichbaum – Apostel Weissbier Dunkel	5.3	Germany
**dw2***	Dunkelweizen	Перша приватна броварня (Persha Pryvatna Brovarnya) – Бочкове Нефільтроване Темне	4.8	Ukraine
**dw3***	Dunkelweizen	Radeberger Gruppe – Schöfferhofer Weizen Dunkel	5.0	Germany
**hw1**	Hefeweizen	Пивоварна 359 (Brewery 359) – Divo Pivo Weiss	4.5	Bulgaria
**hw2***	Hefeweizen	Brau Union Österreich – Edelweiss Weizenbier	5.3	Austria
**hw3***	Hefeweizen	Carlsberg Ukraine – Лев Біле Пшеничне	5.0	Ukraine
**hw4***	Hefeweizen	Radeberger Gruppe – Schöfferhofer Hefeweizen / Weizen	5.0	Germany
**hw5**	Hefeweizen	Pinta – Bawarka	5.7	Poland
**hw6**	Hefeweizen	Jagerhof – Weiss	5.0	Bulgaria
**hw7**	Hefeweizen	Mad Scientist – Monkey Temple	4.6	Hungary
**hw8**	Hefeweizen	Neumarkter Lammsbräu – Weiße	5.1	Germany
**hw9**	Hefeweizen	Schneider Weisse G. Schneider & Sohn – Helle Weisse (TAP01)	4.9	Germany
**hw10**	Hefeweizen	Primátor – Weizen	4.8	Czech Republic
**hw11**	Hefeweizen	Brauhaus Leikeim – Leikeim Weizen	5.4	Germany
**hw12**	Hefeweizen	MONYO Brewing Co. – Schatzi	5.5	Hungary
**hw13**	Hefeweizen	Hills Brewery Bulgaria – Hills Weizen	4.9	Bulgaria
**hw14**	Hefeweizen	La Quince Brewing Co. – Weizenland	5.5	Spain
**hw15**	Hefeweizen	Van Moll – Langharig Tuig	5.5	Netherlands
**kw1**	Kristallweizen	Kulmbacher Brauerei – Kapuziner Weißbier Kristallklar	5.4	Germany
**kw2**	Kristallweizen	Schneider Weisse G. Schneider & Sohn – Kristall (TAP02)	5.3	Germany

**kw3***	Kristallweizen	Radeberger Gruppe – Schöfferhofer Eis–Kristall	4.9	Germany
**wb1**	Weizenbock	Mahrs Bräu – Weisser Bock	7.2	Germany
**wb2**	Weizenbock	Bayerische Staatsbrauerei Weihenstephan – Weihenstephaner Vitus	7.7	Germany
**wb3**	Weizenbock	Schneider Weisse G. Schneider & Sohn / Brooklyn Brewery – Hopfenweisse (TAP05)	8.2	Germany/USA
**wt1**	Witbier	Brouwerij St.Bernardus – Wit	5.5	Belgium
**wt2**	Witbier	Delirium – Huyghe Brewery – Blanche des Neiges	4.9	Belgium
**wt3***	Witbier	Hoegaarden – Wit / Blanche	4.9	Belgium
**wt4**	Witbier	Superfood Beers – LUA – Lucuma / Acerola	4.7	Belgium
**wt5**	Witbier	Brasserie du Bocq – Blanche de Namur	4.5	Belgium
**wt6***	Witbier	Carlsberg Ukraine – Robert Doms Бельгійський (Robert Doms Belgian Style)	4.3	Ukraine
**wt7**	Witbier	LERVIG – Super Blanc	4.7	Norway
**wt8**	Witbier	BrewFist – La Mosca	5.2	Italy
**wt9***	Witbier	Kronenbourg Brewery – 1664 Blanc	5.0	Serbia
**wt10**	Witbier	Van Moll – Nirvana (2023)	5.2	Netherlands
**wt11**	Witbier	Põhjala – Udune	4.5	Estonia
**wt12**	Witbier	PINTA / Brasserie du Pays Flamand – Król Lata	4.0	Poland/France
**wt13**	Witbier	Hills Brewery Bulgaria – Summer Blanche – Session Ale	3.8	Bulgaria
**wt14**	Witbier	Frontaal Brewing Co. – Witte Simmie	5.2	Netherlands
**wt15**	Witbier	Mikkeller – Blanche De Mikkeller	5.0	Denmark

*Commercial beer samples

Table S2. Misclassification table of the OPLS-DA model distinguishing Hefeweizen, Weizenbock and Witbier.

	Members	Correct	Hefeweizen	Weizenbock	Witbier
**Hefeweizen**	21	95.24%	20	0	1
**Weizenbock**	3	100%	0	3	0
**Witbier**	15	100%	0	0	15
**Total**	39	97.44%	20	3	16

Table S3. Misclassification table of the OPLS-DA model distinguishing Dunkelweizen, Kristallweizen and Hefeweizen.

	**Members**	**Correct**	**Dunkelweizen**	**Kristallweizen**	**Hefeweizen**
**Dunkelweizen**	3	100%	3	0	0
**Kristallweizen**	3	100%	0	3	0
**Hefeweizen**	15	100%	0	0	15
**Total**	21	100%	3	3	15

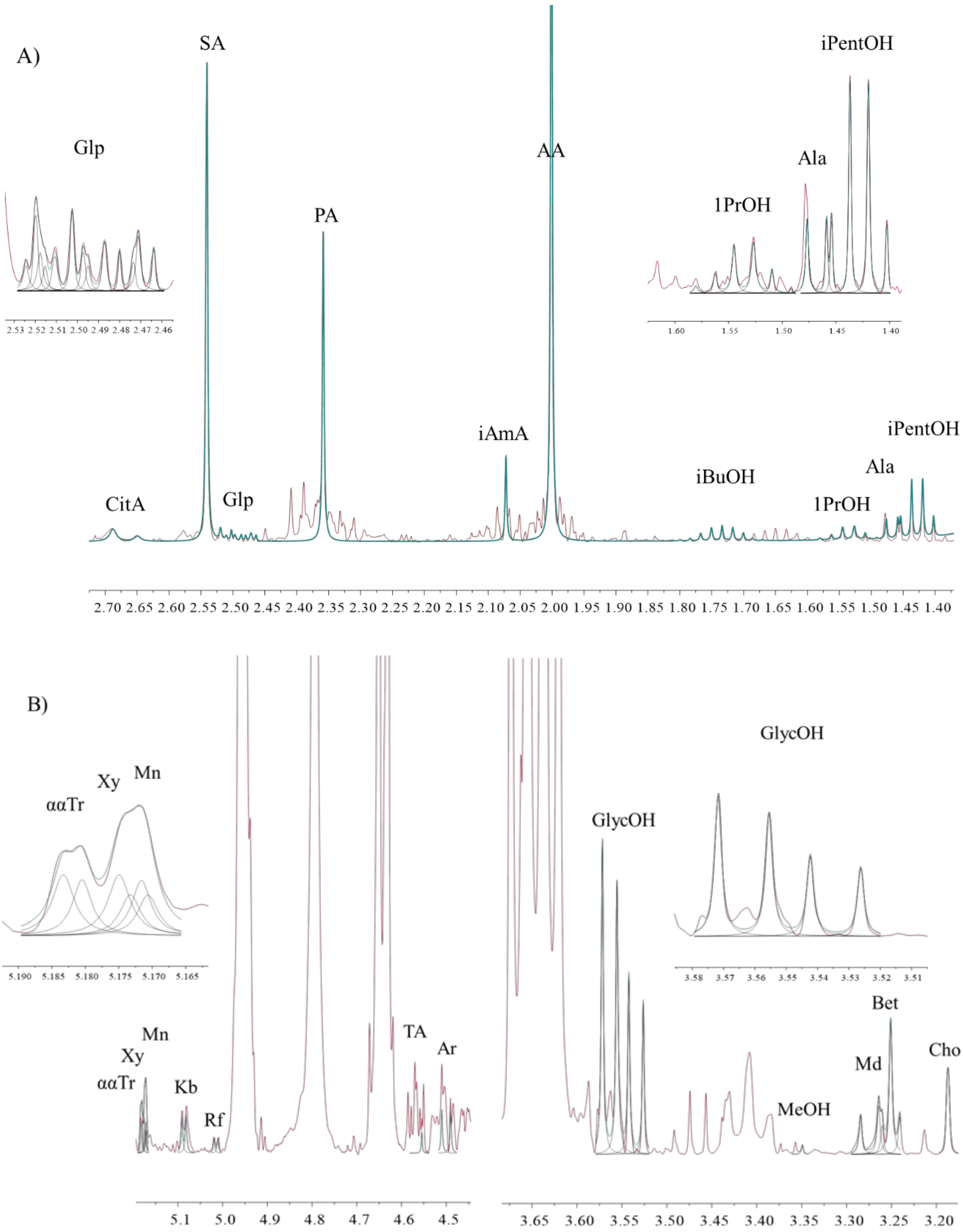
A)
SA
Glp
PA
AA
iPentOH
Ala
1PrOH
iAmA
CitA
iBuOH
2.70 2.65 2.60 2.55 2.50 2.45 2.40 2.35 2.30 2.25 2.20 2.15 2.10 2.05 2.00 1.95 1.90 1.85 1.80 1.75 1.70 1.65 1.60 1.55 1.50 1.45 1.40
B)
ααTr
Xy
Mn
Kb
Rf
TA
Ar
GlycOH
MeOH
Md
Bet
Cho
5.1 5.0 4.9 4.8 4.7 4.6 4.5
3.65 3.60 3.55 3.50 3.45 3.40 3.35 3.30 3.25 3.20

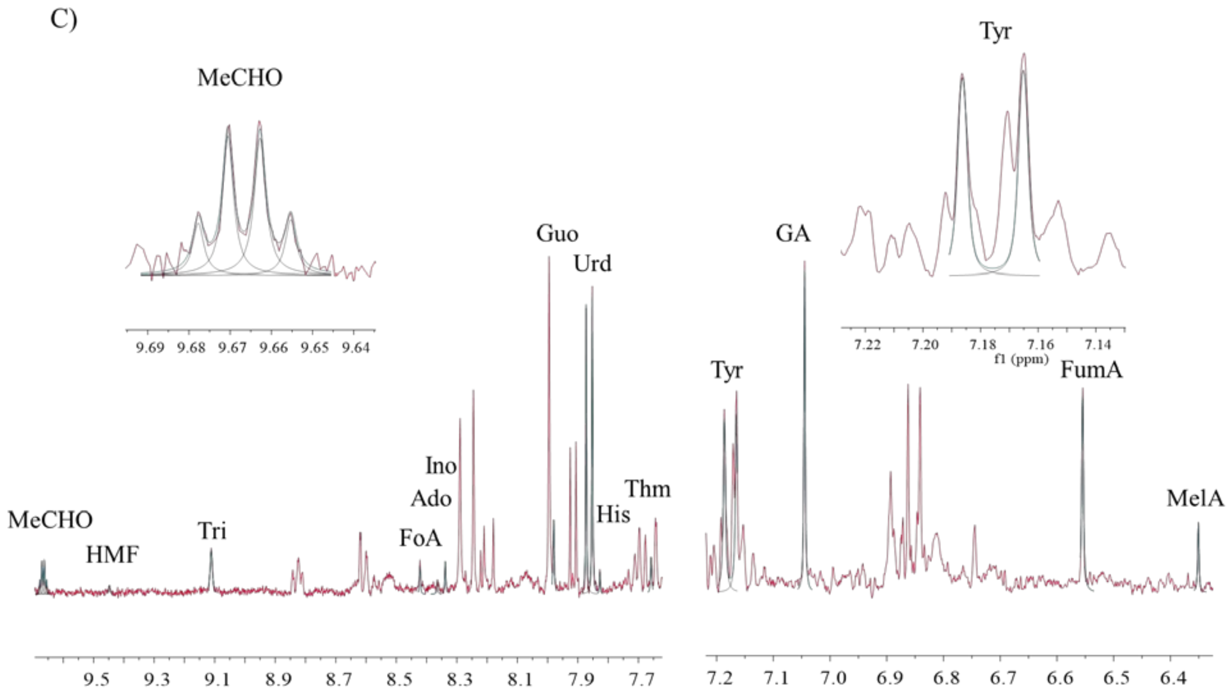

Figure S1. Experimental (dark red) and deconvoluted (teal) ^{1}H NMR spectra of sample **wt10** shown across selected spectral regions: (a) 1.37–2.73 ppm, (b) 3.17–3.68 ppm, 4.44–5.20 ppm, and (c) 6.32–7.21 ppm, 7.62–9.70 ppm. Only signals used for quantitative NMR analysis were subjected to deconvolution.

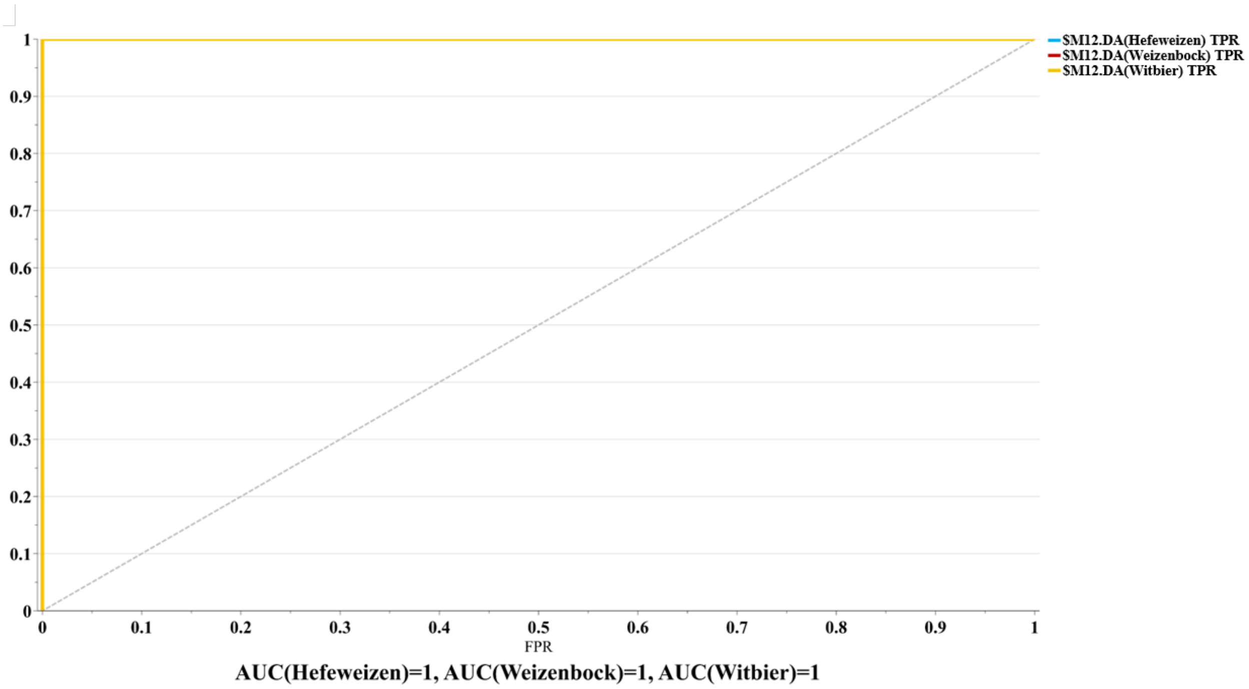

Figure S2. ROC curve illustrating the OPLS-DA model's classification accuracy in distinguishing Hefeweizen, Weizenbock and Witbier.

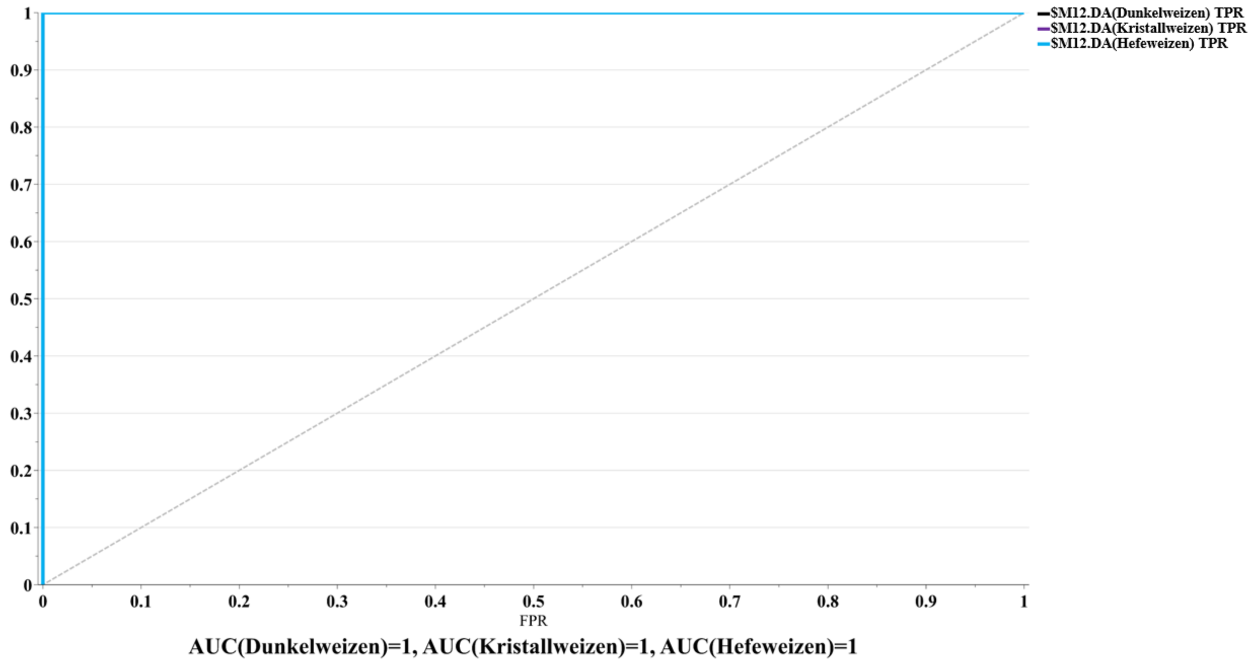

Figure S3. ROC curve illustrating the OPLS-DA model's classification accuracy in distinguishing Dunkelweizen, Kristallweizen and Hefeweizen.

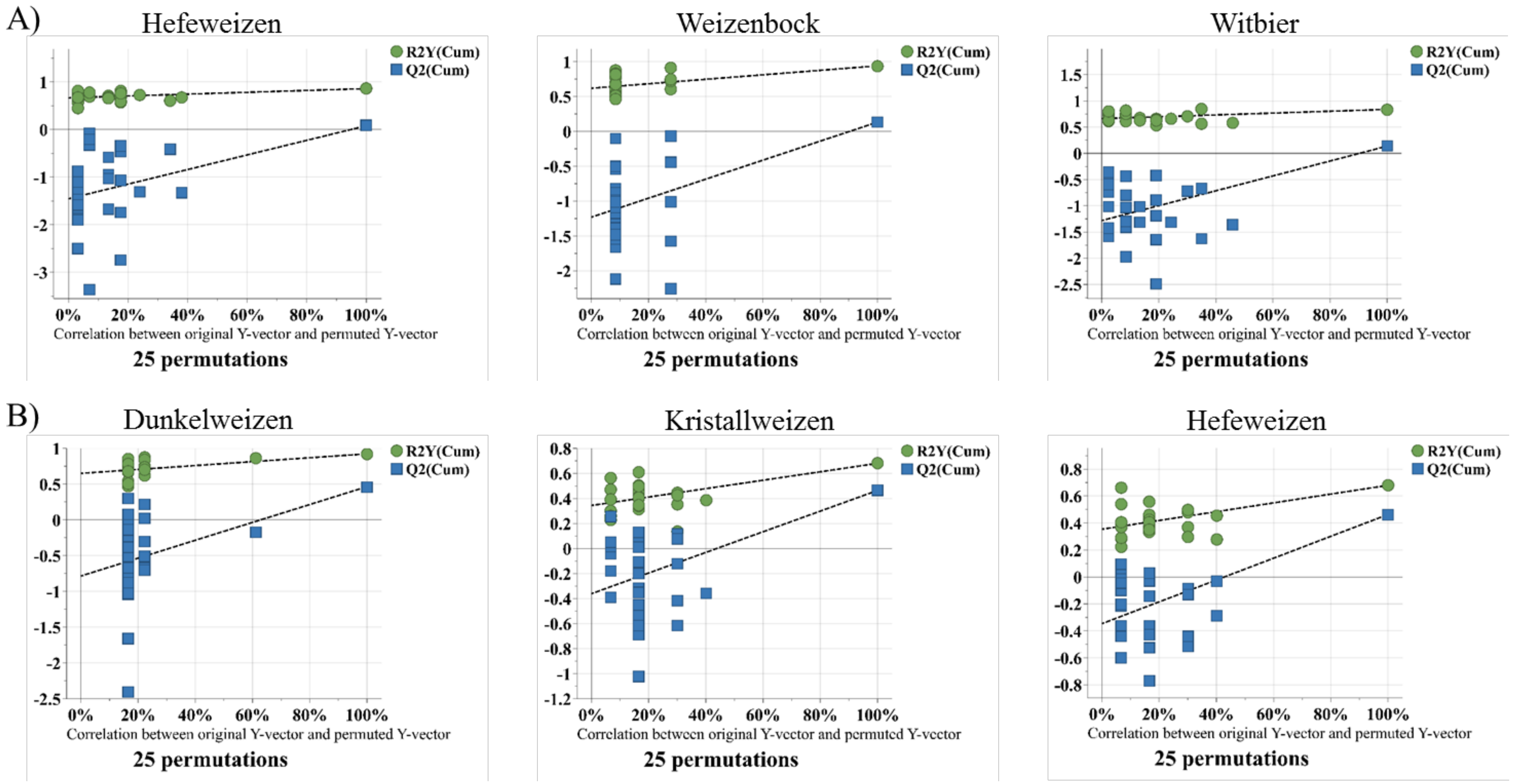

Figure S4. Permutation tests (25 iterations) assessing the statistical significance and robustness of two OPLS-DA models: (a) discrimination of three wheat beer styles – Hefeweizen, Weizenbock, Witbier and (b) differentiation of three wheat beer substyles – Dunkelweizen, Kristallweizen and Hefeweizen. The results confirm valid and non-random class separation in both models.

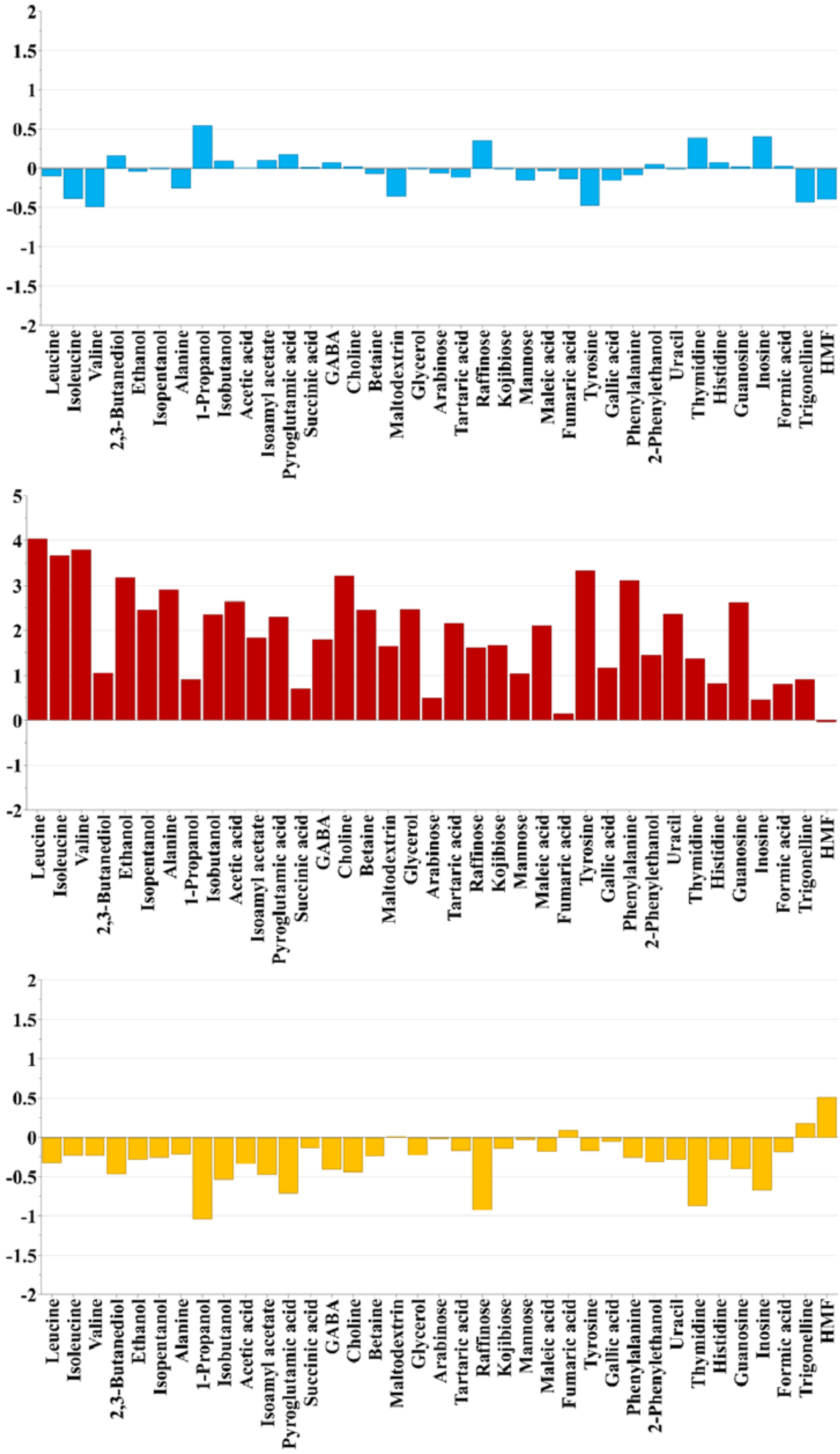

Figure S5. OPLS-DA contribution plots demonstrating the differentiation of significant compounds among Hefeweizen (blue), Weizenbock (red) and Witbier (yellow).

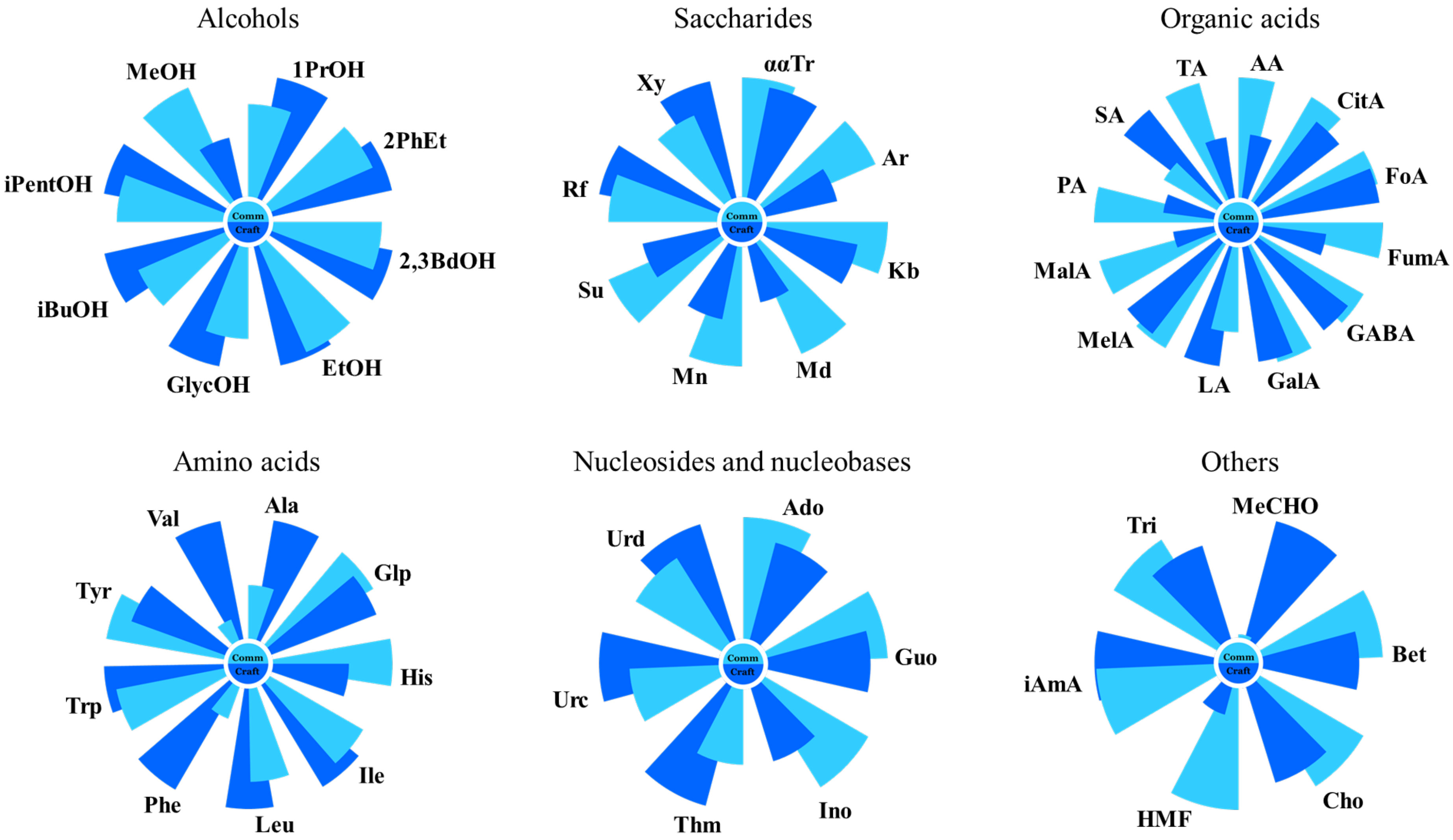

Figure S6. Nightingale diagrams illustrating the levels of metabolites in commercial (light blue) and craft (blue) Hefeweizen samples, categorized by compound class (alcohols, saccharides, organic acids, amino acids, nucleosides, and others).

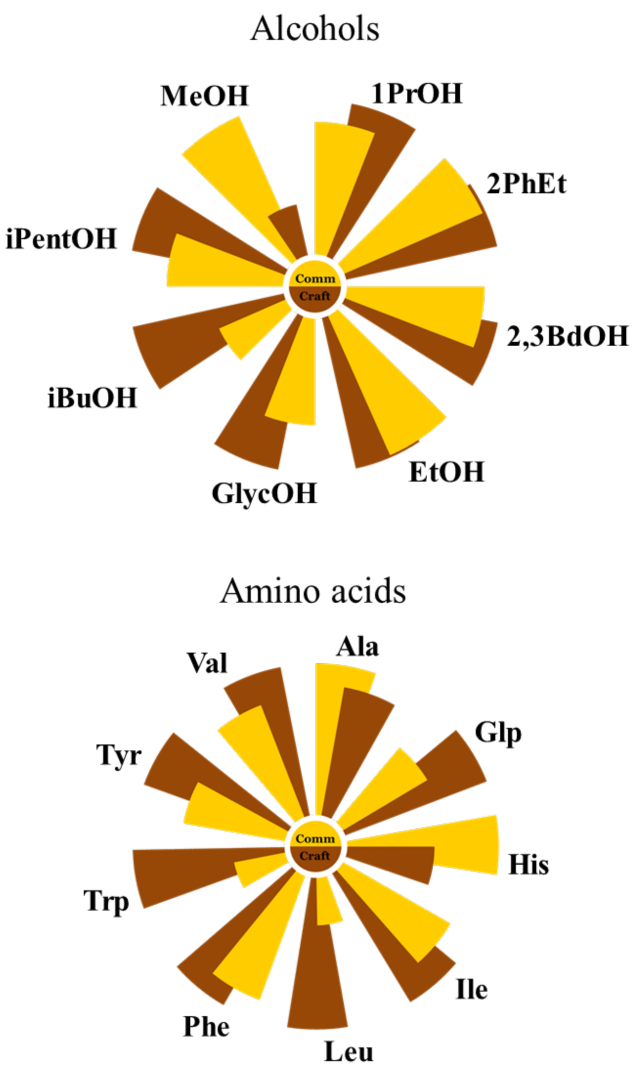

Figure S7. Nightingale diagrams illustrating the levels of metabolites in commercial (yellow) and craft (brown) Witbier samples, categorized by compound class (alcohols, saccharides, organic acids, amino acids, nucleosides, and others).

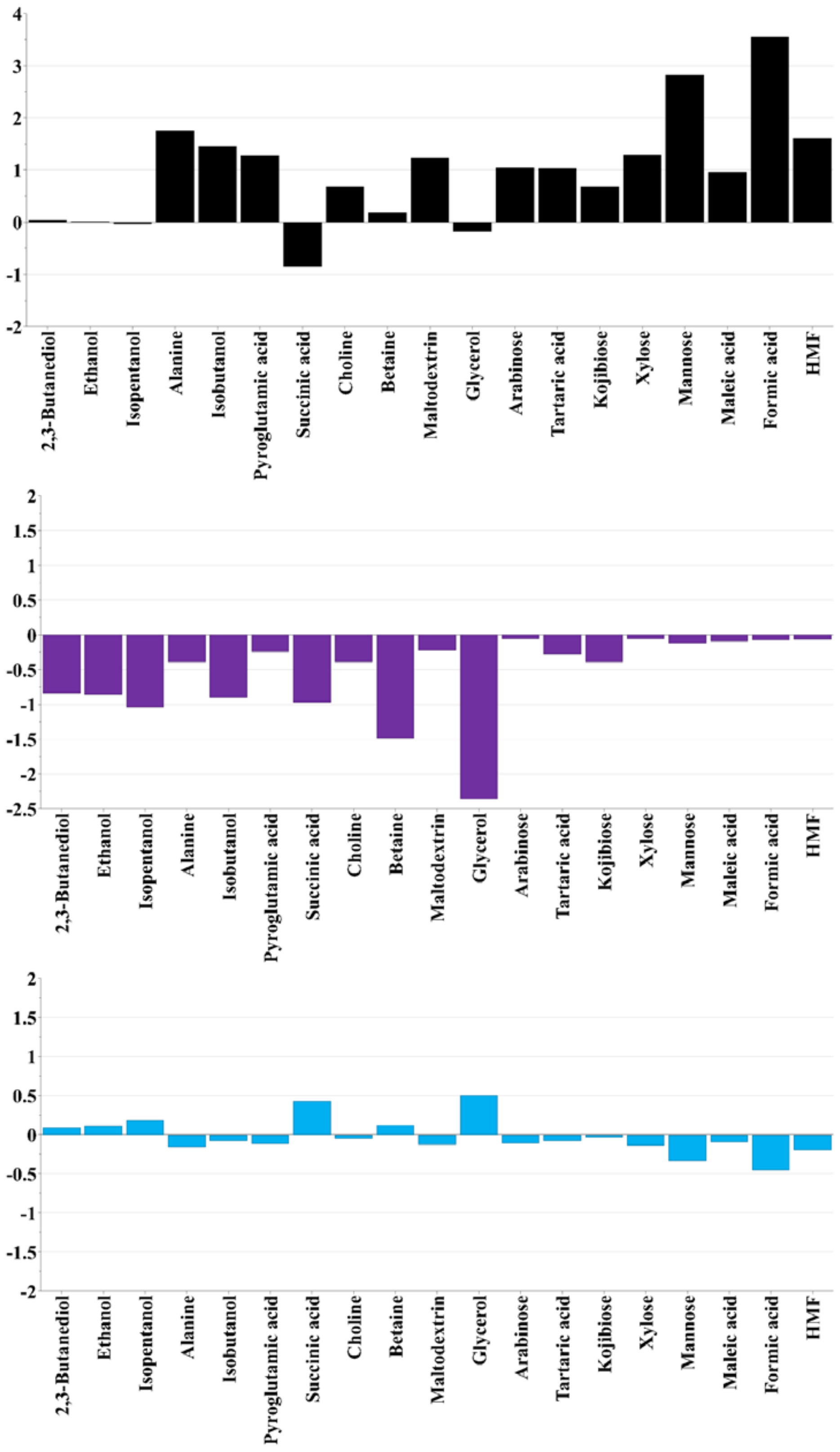

Figure S8. OPLS-DA contribution plots demonstrating the differentiation of significant compounds among Dunkelweizen (black), Kristallweizen (purple) and Hefeweizen (blue).
